# Sodium-Glucose Cotransporter-2 (SGLT2) Inhibitor Therapy for the Primary and Secondary Prevention of Heart Failure in Patients With and Without Type 2 Diabetes Mellitus: A Systematic Review

**DOI:** 10.7759/cureus.37388

**Published:** 2023-04-10

**Authors:** Maureen Wahinya, Zahid Khan

**Affiliations:** 1 Internal Medicine, Kenyatta University Teaching, Referral and Research Hospital, Nairobi, KEN; 2 Acute Medicine, Mid and South Essex NHS Foundation Trust, Southend-on-Sea, GBR; 3 Cardiology, Bart’s Heart Centre, London, GBR; 4 Cardiology and General Medicine, Barking, Havering and Redbridge University Hospitals NHS Trust, London, GBR; 5 Cardiology, Royal Free Hospital, London, GBR

**Keywords:** awareness of cardiovascular disease, heart failure with reduced ejection fraction, heart failure with preserved ejection fraction (hfpef), cardiovascular prevention, hypoglycaemia, randomized clinical trial, renal impairment, sglt-2 inhibitor, diabetes mellitus type 2, heart failure

## Abstract

The global prevalence of heart failure (HF) is rising and carries a heavy social and economic burden. Type 2 diabetes mellitus (T2DM) patients are at an increased risk of incident HF even in the absence of cardiovascular risk factors. Patients with established HF are at an increased risk of death following a worsening HF event. Various trials on sodium-glucose cotransporter-2 (SGLT2) inhibitors have shown that these novel drugs prevent incident HF and reduce the risk of worsening HF in both patients with T2DM and those without diabetes. This literature review analyzed the data from 13 randomized controlled trials that met the pre-specified inclusion criteria. The aim was to compare the clinical outcomes of SGLT2 inhibitors for primary and secondary prevention of HF in patients with T2DM and those without diabetes. In addition, this study collated and summarized the patients’ clinical characteristics with respect to the clinical outcome, and finally, it evaluated the safety considerations when using SGLT2 inhibitors. The data showed that SGLT2 inhibitors are effective and safe in the primary and secondary prevention of HF across a broad spectrum of patient populations and care settings. Therefore, wider eligibility for their use should be considered.

## Introduction and background

Heart failure (HF) is the final common pathway for most heart diseases. Global prevalence and incidence have been on the rise, more so in developing nations [1]. HF carries a heavy social and economic burden. For example, in the United States of America, $346.17 billion is spent annually managing HF [2]. The development of HF portends a poor survival prognosis, with nearly half of the patients dying within five years following diagnosis [3]. In patients with type 2 diabetes mellitus (T2DM), HF is the second most prevalent cardiovascular disease (CVD) after atherosclerosis CVD [4]. These patients have a nearly two-fold increased risk of developing new-onset HF irrespective of cardiovascular (CV) risk factors [5]. Similarly, chronic kidney disease (CKD) is strongly linked to the development of HF and with half of CKD patients exhibiting CVD [6,7]. In patients with existing HF, regardless of T2DM status, the primary focus has been preventing hospitalization from worsening HF and possibly death [8]. Despite previous HF therapies resulting in the improvement of the prognosis of HF patients, this benefit was majorly seen in patients with HF with reduced ejection fraction (HFrEF) while the options for patients with HF with a preserved ejection fraction (HFpEF) remained limited [8,9].

The hypoglycemic drugs, sodium-glucose cotransporter-2 (SGLT2) inhibitors, have emerged as formidable drugs demonstrating primary and secondary prevention of HF events in both T2DM and non-diabetes mellitus (DM) patients [8,10]. Some postulated mechanisms of action of SGLT2 inhibitors that are beneficial in HF include their natriuresis and osmotic diuretic effects, which reduce the preload and afterload in HF [11], reduce the blood pressure [12,13], improve the cardiac metabolism and overall performance [14], preventing cardiac remodeling [15-17], and increase in erythropoietin levels with consequent improvement in myocardial tissue oxygenation [18].

This study will synthesize the available evidence on the efficacy of SGLT2 inhibitors for the primary and secondary prevention of HF in both T2DM and non-DM patients. In addition, it will help come up with an algorithm that will aid clinicians in determining which patients would benefit from and can be safely initiated on an SGLT2 inhibitor for purposes of HF prevention based on the patient’s clinical characteristics and clinical parameters, as evidenced in clinical trials.

## Review

Methodology

A systematic literature search using Google Scholar, PubMed, Cochrane Library, MEDLINE, Embase, Findit, and ScienceDirect databases was done for studies that were relevant to this narrative literature review. The keywords used for the literature search were related to SGLT2 inhibitors (“dapagliflozin,” “empagliflozin,” “canagliflozin,” “ertugliflozin,” and “sotagliflozin”), “heart failure” (“HFrEF” and “HFpEF), “type 2 diabetes,” and “cardiovascular and renal outcome with SGLT2 inhibitors.” The inclusion criteria for this review were limited to randomized control trials (RCTs) published between 2012 and 2022 and in the English language.

**Figure 1:**
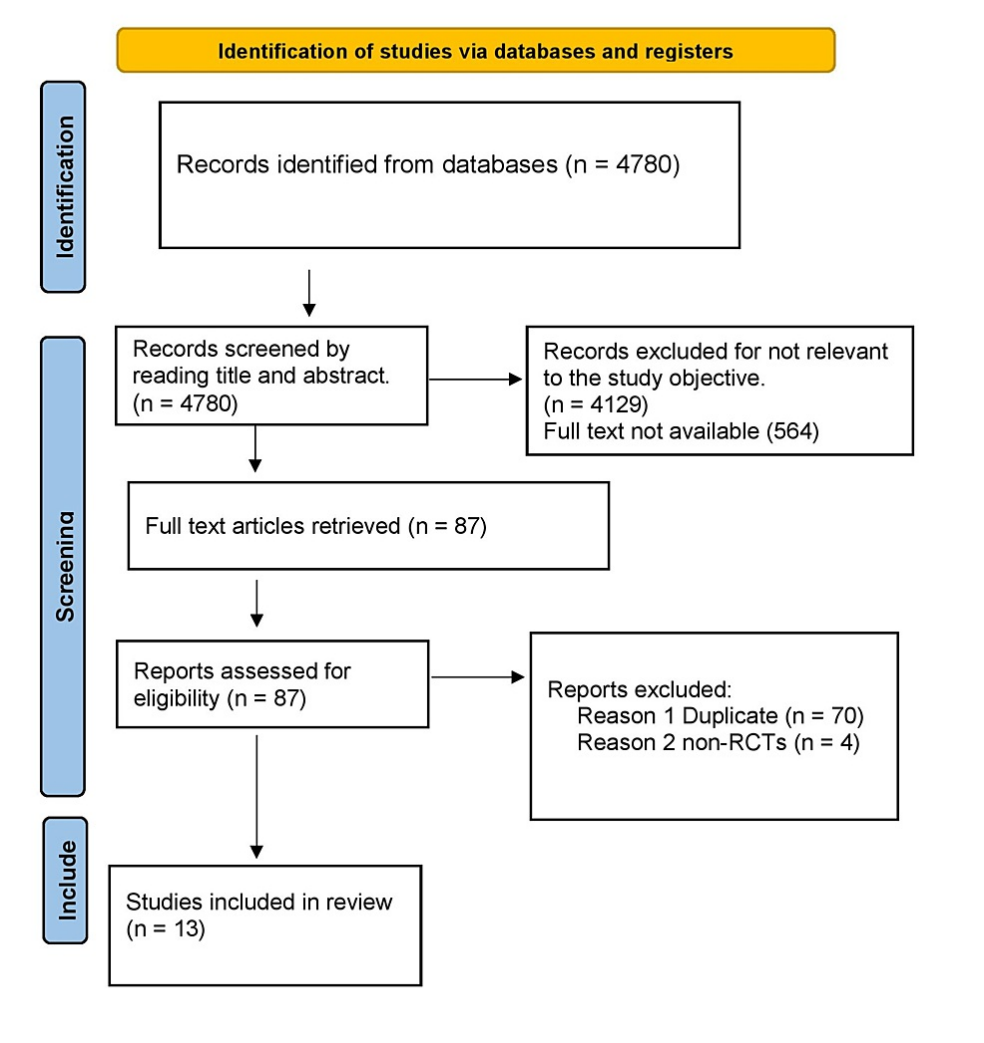
Preferred Reporting Items for Systematic Reviews and Meta-Analyses chart

A total of 13 studies were included in this literature review, as shown in the PRISMA chart (Figure 1).

Results and discussion

Eight studies on SGLT2 inhibitors for the primary prevention of HF (Table 1) and five studies on the secondary prevention of HF (Table 2) were included.

**Table 1:** Outcome studies on primary prevention of heart failure T2DM: type 2 diabetes mellitus; CVD: cardiovascular disease; CV: cardiovascular; CKD: chronic kidney disease; MACE: major adverse cardiac events; HHF: hospitalization for heart failure.

Trials	SGLT2 inhibitor	Population	Participants	Follow-up	Primary outcome
EMPAREG OUTCOME [19]	Empagliflozin	T2DM with CVD	7,020	3.1 years	Reduced MACE (HR: 0.86, 95% CI: 0.74-0.99)
CANVAS PROGRAM [20]	Canagliflozin	T2DM with increased CV risk	10,142	3.9 years	Reduced MACE (HR: 0.86, 95% C: 0.75-0.97)
DECLARE TIMI 58 [21]	Dapagliflozin	T2DM with CVD or CV risk factors	17,160	4.2 years	Reduced MACE, CV death, or HHF (HR: 0.95, 95% CI: 0.84-1.03)
VERTIS-CV [22]	Ertugliflozin	T2DM with CVD	8,246	3.5 years	Reduced MACE (HR: 0.97, 95% CI: 0.85-1.11)
CREDENCE [23]	Canagliflozin	T2DM CKD patients	4,401	2.6 years	Reduced composite outcome of renal events (HR: 0.70, 95% CI: 0.59-0.82)
DAPA-CKD [24]	Dapagliflozin	CKD patients irrespective of T2DM	4,304	2.4 years	Reduced composite outcome of renal events (HR: 0.61, 95% CI: 0.51-0.72)
SCORED [25]	Sotagliflozin	T2DM CKD patients	10,584	16 months	Reduced cumulative deaths from CVD, HHF, & need for HF visit (HR: 0.74, 95% CI: 0.63-0.88)
EMPA-KIDNEY [26]	Empagliflozin	CKD patients irrespective of T2DM	6,609	2 years	Reduced initial occurrence of renal deterioration or CV death (HR: 0.72, 95% CI: 0.64-0.82)

**Table 2:** Outcome studies on secondary prevention of heart failure SGLT2: sodium-glucose cotransporter-2; HF: heart failure; HHF: hospitalization for heart failure; T2DM: type 2 diabetes mellitus; DM: diabetes mellitus; CV: cardiovascular.

Trial	SGLT2 inhibitor	Population of HF patients	Number of patients	Follow-up	Primary outcome	Worsening HF	HHF
DAPA-HF [27]	Dapagliflozin	T2DM 42%, no DM 58%	4,744	1.5 years	Reduced combination of worsening HF & CV death (HR: 0.74, 95% CI: 0.65-0.85)	HR: 0.70, 95% CI: 0.59-0.83	HR: 0.70, 95% CI: 0.59-0.83
EMPORER REDUCED [28]	Empagliflozin	T2DM 50%, no DM 50%	3,730	1.3 years	Reduced combination of HHF & CV death (HR: 0.75, 95% CI: 0.65-0.86)	-	HR: 0.69, 95% CI: 0.59-0.81
EMPEROR PRESERVED [29]	Empagliflozin	T2DM 49%, no DM 51%	5,988	2.2 years	Reduced combination of HHF & CV death (HR: 0.79, 95% CI: 0.69-0.90)	-	HR: 0.60-0.83
SOLOIST HF [30]	Sotagliflozin	T2DM 100%	1,222	9 months	Reduced sum of CV deaths, HHF, & urgent HF visits (HR: 0.67, 95% CI; 0.52-0.85)	HR: 0.64, 95% CI: 0.49-0.83	-
DELIVER [31]	Dapagliflozin	T2DM 45%, no DM 55%	6,263	2.3 years	Reduced combination of CV or worsening HF (HR: 0.82, 95% CI: 0.73-0.92)	HR: 0.79, 95% CI: 0.69-0.91	HR: 0.77, 95% CI: 0.67-0.89

SGLT2 inhibitors for the primary prevention of HF in T2DM patients

Following the cardiovascular outcome trials (CVOTs) of SGLT2 inhibitors, these drugs were serendipitously shown to confer a reduction of HF events among T2DM patients, a finding that was independent of their glucose-lowering effect. Data show that SGLT2 inhibitors result in a moderate reduction in the incidence of major adverse cardiac events (MACE); however, their strongest and most consistent benefit is in the reduction of incident HF. Notably, the overwhelming majority of the patients had no history of HF; hence evidence on primary prevention of HF was apparent. These findings were further confirmed in a meta-analysis by Kaze et al., where the pooled effect size of SGLT2 inhibitors from data of the CVOTs and the renal outcome trials found that using an SGLT2 inhibitor lowered the risk of MACE by 17% (I2 = 33.8%, P = 0.183) and substantially lowered the risk of hospitalization for heart failure (HHF) by 38% (I2 = 0%, P = 0.844) [32].

The magnitude of the effect in the reduction of HHF was an unexpected but welcome discovery, as no previous CVOT of a hypoglycemic drug had demonstrated a reduction in the incidence of HF.

SGLT2 inhibitors also demonstrated a compelling and significant role in the primary prevention of HF in patients without pre-existing CVD. This was first evident from the DECLARE TIMI 58 trial, which recruited the largest number of individuals free from CVD but exhibiting multiple CV risk factors. However, this study did not demonstrate a significant reduction in MACE; a finding that could suggest that SGLT2 inhibitors are more efficacious in the prevention of MACE in individuals with a greater CVD risk or prior history of CV events. The data from CREDENCE, DAPA-CKD, and SCORED trials further supported this evidence of HF prevention in patients without CVD as these trials also enrolled a significant percentage of people without CVD. This is an important finding because DM patients are at increased risk of incident HF even without established CVD [33].

In a systematic review and meta-analysis of data from EMPAREG OUTCOME, CANVAS program, and DECLARE TIMI 58 trials, SGLT2 inhibitors lowered the risk of HHF by 31%, and this risk reduction was maintained regardless of the presence of atherosclerotic cardiovascular disease (ASCVD) [34]. SGLT2 inhibitors significantly reduced the risk of HHF in patients with ASCVD and marginally in those without ASCVD. The risk of HHF was reduced by 29% in patients with pre-existing HF and 21% in patients without HF. Findings from this meta-analysis further support the data that SGLT2 inhibitors appear to have a greater significance in the reduction of HHF than in the reduction of MACE.

Renal outcomes trials with SGLT2 inhibitors [16-18] also support this consistent risk reduction in HHF. The DAPA-CKD study and the SCORED study showed that even in patients with a reduced estimated glomerular filtration (eGFR) as low as 25 ml/min/1.73 m^2^, SGLT2 inhibitors still conferred the benefit of a reduction in HHF without worsening renal function. The data from these trials show a relationship between baseline renal function and a decrease in HHF. SGLT2 inhibitors resulted in a greater risk reduction of HHF in patients with a worse baseline renal function without renal function deterioration. Instead, SGLT2 inhibitors reduced the composite of renal endpoints (deterioration of renal function, advancement to end-stage renal disease, and mortality from renal causes). This is an important finding because the risk of HF increases with a declining eGFR [35]; therefore, these data give confidence in the role of SGLT2 inhibitors in renoprotection as well as in the reduction of incident HF in T2DM. However, the EMPA-KIDNEY study was the only one that failed to show a significant benefit in the prevention of HHF. This was explained by the lower occurrence of CV events, which reduced the study’s power to assess the secondary outcomes.

SGLT2 inhibitors for the primary prevention of HF in patients without diabetes

**Figure 2:**
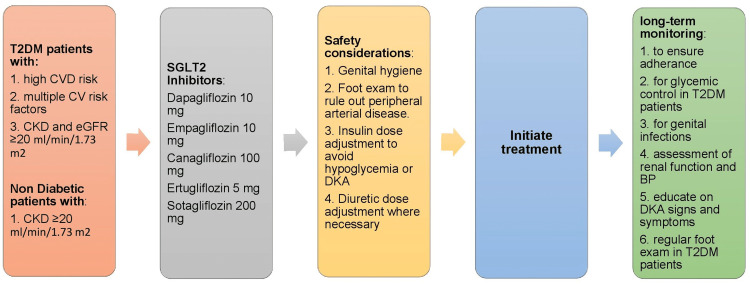
Algorithm for initiating SGLT2 inhibitors for the primary prevention of heart failure SGLT2: sodium-glucose cotransporter-2; T2DM: type 2 diabetes mellitus; CVD: cardiovascular disease; CV: cardiovascular; CKD: chronic kidney disease; eGFR: estimated glomerular filtration; DKA: diabetic ketoacidosis; BP: blood pressure.

The only tangible evidence of a possible benefit for the primary prevention of HF with SGLT2 inhibitors in non-diabetic patients comes from the DAPA-CKD study [24]. In the DAPA-CKD study, about 32% of the patients were not diabetic. Data from the subgroup analysis of the primary outcome showed that dapagliflozin conferred benefits in both T2DM and non-DM patients. Likewise, in the prespecified secondary outcome of CV death or HHF, dapagliflozin reduced the risk in both patient groups as evidenced by a P interaction of 0.78 [36]. This finding is exciting because it gives insight into the potential role of SGLT2 inhibitors for the primary prevention of HF in non-DM patients, specifically those with non-DM CKD. However, because these data were from a subgroup analysis of the secondary endpoint, the data were not powered to make any conclusions. Given that HF is the leading cause of CVD among CKD patients [37], the potential role of SGLT2 inhibitors in preventing incident HF in non-DM CKD patients will be a game changer. Figure 2 shows the algorithm for initiating SGLT2 inhibitors in primary heart failure patients.

SGLT2 inhibitors for the secondary prevention of HF in both T2DM and non-diabetic patients with HFrEF

The DAPA-HF study [27] and the EMPEROR-Reduced study [28] investigated the efficacy of dapagliflozin and empagliflozin, respectively, in patients with HFrEF. Specifically, these trials examined whether SGLT2 inhibitors reduced the combined risk of CV mortality and HF events in patients with HFrEF defined as a left ventricle ejection fraction (LVEF) ≤ 40%. Both studies convincingly demonstrated that SGLT2 inhibitors reduced the incidence of HF events in patients with HFrEF when added to standard care. The DAPA-HF study showed that dapagliflozin reduced the combined primary endpoint of CV mortality or worsening HF by 26%. In comparison, the EMPEROR-Reduced study showed a 25% relative risk reduction with empagliflozin for a combined primary endpoint of CV mortality or HHF. This reduction was consistent and significant in both studies regardless of DM status. Notably, most patients in both trials did not have diabetes. The number needed to treat (NNT) to prevent an event was 20 in the DAPA-HF study for a median duration of treatment of 18 months, and NNT of 19 in the EMPEROR-Reduced study for a median duration of treatment of 16 months.

There was an almost similar reduction in the incidence of HHF in both trials. Interestingly, the EMPEROR-Reduced study did not show a significant reduction in the incidence of CV mortality. This finding could be explained by a much sicker population in the EMPEROR-Reduced study compared to the DAPA-HF study.

**Figure 3:**
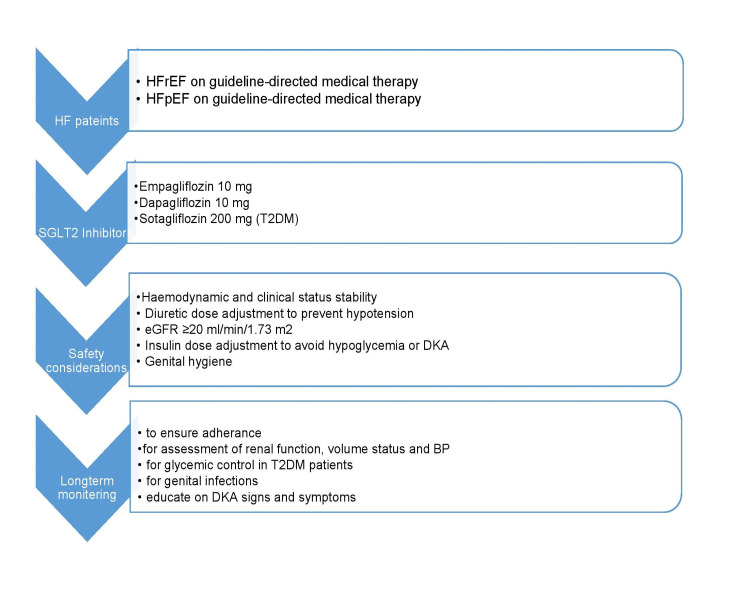
Algorithm for initiating SGLT2 inhibitors for the secondary prevention of heart failure SGLT2: sodium-glucose cotransporter-2; HF: heart failure; HFrEF: heart failure with reduced ejection fraction; HFpEF: heart failure with a preserved ejection fraction; T2DM: type 2 diabetes mellitus; eGFR: estimated glomerular filtration; DKA: diabetic ketoacidosis; BP: blood pressure.

The SOLOIST-WHF study had a majority of the patients with an LVEF < 50% [30]. This study was unique because it included patients in a different care setting than the previous studies. Sotagliflozin was initiated in patients with a recent episode of decompensating HF, unlike in the other trials where patients had stable chronic HF. Nevertheless, despite being terminated early and with a change in the initial primary endpoint, treatment with sotagliflozin resulted in an overall lower number (31% relative risk reduction) of CV deaths, HHF, and urgent HF hospital visits. Notably, these encouraging results were obtained in the background of the high use of guideline-directed medical therapy. As worsening HF is of prognostic value in patients with existing HF, the results of these trials have proved to be crucial in the secondary prevention of HF and, importantly, in lowering the risk of death. Evidence from these three studies led to these SGLT2 inhibitors being included in the newly revised 2021 European Society of Cardiology (ESC) HF clinical guidelines [8] as part of HF therapies with a class 1 indication. Sotagliflozin is, however, only considered in T2DM with HFrEF. Figure 3 shows the algorithm for the use of SGLT2 inhibitors for secondary prevention in patients with heart failure.

SGLT2 inhibitors for the secondary prevention of HF in both T2DM and non-diabetic patients with HFpEF and HFmrEF

The two landmark trials on SGLT2 inhibitors on HFpEF, the EMPEROR-Preserved [29] and the DELIVER trial [31], examined the efficacy of empagliflozin and dapagliflozin, respectively, in patients with HFpEF irrespective of diabetes status. The first of the studies was the EMPEROR-Preserved, which showed that empagliflozin significantly reduced the risk of the primary endpoint (CV death or HHF) by 21% in both T2DM and non-diabetic patients, and the effect of empagliflozin had no interaction with diabetes status. The NNT to prevent an event was 31 after a median duration of treatment of 26 months.

Despite this study being described as the “first positive trial in HFpEF,” it did not seem to confer any significant reduction in CV mortality and in overall mortality. This study was also not strictly restricted to patients with HFpEF. It recruited patients with an LVEF > 40%, which, according to the 2021 ESC definition, encompasses both HF with mildly reduced ejection fraction (HFmrEF) (LVEF = 40-49%) and HFpEF (LVEF ≥ 50%) [8]. However, in the prespecified subgroup analysis, the effect of empagliflozin on the primary endpoint was consistent across all the LVEF categories, but the efficacy was shown to attenuate with increasing LVEF categories (HR = 0.71 in LVEF < 50%, HR = 0.80 in LVEF ≥50-<60%, and HR = 0.87 in LVEF ≥ 60%). Additionally, when compared to the studies on HFrEF [20,21,23], empagliflozin failed to show any significant benefit on the composite of renal endpoints (HR = 0.95, 95% CI = 0.73-1.24); except in a significant reduction in the rate of decline in eGFR (HR = 1.36, 95% CI = 1.06-1.66). Therefore, interpretation of these results should be done cautiously in patients with pure HFpEF.

The DELIVER study is the largest and most recent trial of SGLT2 inhibitors in patients with HFpEF and HFmrEF. Dapagliflozin led to an 18% reduction in the composite outcome of CV death or worsening HF with no interaction of diabetes status. The NNT to prevent an event was 32 after a median duration of treatment of 2.3 years. Although the DELIVER study was not powered to assess dapagliflozin’s effect on CV mortality, the results indicated that dapagliflozin did not reduce CV death or overall mortality, but did show benefit in the reduction of HHF by 23%. Similar to the EMPEROR-Preserved study, this study recruited patients with an LVEF > 40% and therefore included patients with HFmrEF (LVEF = 40-49%) as well as those with HFpEF (≥50%). However, unlike its predecessor, the EMPEROR-Preserved study, the DELIVER study did not show any attenuation in benefits with dapagliflozin with increasing LVEF (HR = 0.87 with LVEF ≤ 49%, HR = 0.79 with LVEF = 50-59%, and HR = 0.78 with LVEF ≥ 60%).

In the SOLOIST-WHF study, sotagliflozin was also reported to be significant in the reduction of HHF in patients with HFpEF (HR = 0.48, 95% CI = 0.27-0.86). But because of the small number of patients with HFpEF, there was no definitive recommendation. Just like in the EMPEROR-Preserved study, the SOLOIST-WHF study did not show a reduction in CV mortality or overall mortality. Disappointingly, these three SGLT2 inhibitors did not result in a mortality reduction in patients with HFpEF. Therefore, there is currently no therapeutic drug that confers a reduction in mortality in patients with HFpEF. This uncertainty is conveyed in clinical guidelines where diuretics are the only therapy with a class 1 recommendation for the management of patients with HFpEF. Currently, SGLT2 inhibitors are not recommended as guideline-directed medical therapy for HFpEF [8]. However, with the additional convincing results from the DELIVER trial, we await to see if SGLT2 inhibitors will be recommended in the next review of clinical guidelines.

A meta-analysis of the EMPEROR-Preserved and the DELIVER trial done by Vaduganathan et al. [38] showed that SGLT2 inhibitors not only significantly reduced the combined outcome of CV mortality and HHF, but that they also resulted in the reduction of the individual components, i.e., a 12% reduction in CV death (95% CI = 0.77-1.0) and 26% reduction in HHF (95% CI = 0.67-0.83). However, there was no significant benefit in the reduction of overall mortality. In the same meta-analysis, when all five studies on SGLT2 inhibitors and HF were incorporated, treatment with an SGLT2 inhibitor resulted in a 23% relative risk reduction in the composite endpoint of CV mortality or HHF. This translated to an NNT of 25 to prevent an event over a weighted treatment duration of 23 months. Equally, SGLT2 inhibitors resulted in a 28% relative risk reduction for HHF (NNT of 28), a 13% reduction in the incidence of CV death (NNT of 88), and a 14% reduction in overall mortality (NNT of 92). This benefit was consistent irrespective of diabetes status, care setting, and across all LVEF categories up to LVEF ≤ 60%. Therefore, this shows that SGLT2 inhibitors are beneficial in the secondary prevention of HF in both T2DM and the non-DM population, irrespective of HF classification and care setting.

Clinical characteristics and patient demographics in the studies of primary prevention of HF

Most of the participants’ clinical characteristics were fairly similar, with only a few exceptions (shown in Table 3 below).

**Table 3:** Patients' clinical characteristics from studies on primary prevention of heart failure T2DM: type 2 diabetes mellitus; CVD: cardiovascular disease; CV: cardiovascular; HF: heart failure; HbA1c: glycosylated hemoglobin; eGFR: estimated glomerular filtration rate; SGLT2: sodium-glucose cotransporter-2.

Patient characteristics	EMPAREG OUTCOME [19]	CANVAS [20]	DECLARE TIMI 58 [21]	VERTIS-CV [22]	CREDENCE [23]	DAPA-CKD [24]	SCORED [25]	EMPA-KIDNEY [29]
Mean age (years)	63	63	64	64	62	61	69 (median)	64
Gender (male)	71%	65%	63%	70%	66%	68%	55%	67%
Ethnicity								
White	72%	78%	79%	87%	66%	50%	83%	58%
Black	5.1%	3.0%	3.4%	3.0%	5.1%	4.8%	3.3%	3.9%
T2DM	100%	100%	100%	100%	100%	67%	100%	46%
Existing CVD	99%	72%	40%	100%	50%	37%	-	26%
CV risk factors	-	N/A	60%	-	N/A	N/A	-	N/A
Presence of HF	10%	14%	10%	24%	14%	10%	31%	N/A
Mean HbA1c	8%	8.3%	8.3%	8.2%	8.3%	N/A	8.3% (median)	N/A
Duration of T2DM (mean)	>10 years	13 years	11 years	13 years	N/A	N/A	N/A	N/A
Mean eGFR ml/min/1.73 m2 (SD)	74 (20)	76 (20)	85 (16)	76 (21)	56 (18)	43 (12)	44 (37.0–51.3)	37 (15)
BMI	30	32	32	32	31	29	31	29
Albuminuria								
<30	59.4%	69.8%	67.9%	N/A	0.7%	N/A	35.2%	20.1%
30-<300	28.7%	22.6%	23.4%	N/A	11.3%	N/A	33.4%	28.1%
≥300	11%	7.6%	6.8%	N/A	88%	N/A	31.3%	51.8%
SGLT2 inhibitor daily dose	Empagliflozin 10 mg or 25 mg	Canagliflozin 100 mg-300 mg	Dapagliflozin 10 mg	Ertugliflozin 5 mg or 15 mg	Canagliflozin 100 mg	Dapagliflozin 10 mg	Sotagliflozin 200 mg-400 mg	Empagliflozin 10 mg

The vast majority of the patients in each trial were white males. Most of the trials recruited only T2DM patients except the DAPA-CKD and the EMPA-KIDNEY trials. Most patients had a longstanding history of T2DM with a median duration of ≥10 years. Additionally, the mean glycosylated hemoglobin (HbA1c) in most studies was about 8% to 8.3%, reflecting poor glycemic control. Only a small percentage of patients had a prior history of HF.

The majority of patients in the EMPEREG OUTCOME, CANVAS, and VERTIS-CV trials had established CVD. The DECLARE-TIMI 58 study had the lowest number of patients with existing CVD but the highest number of patients with multiple CV risk factors. Regarding the overall CV and renal risk, patients in the DECLARE-TIMI 58 study harbored the lowest risk, with only 41% of patients having established CVD. Additionally, the patients in the DECLARE-TIMI 58 study had fairly good renal function compared to the other studies. Only 7.4% of the patients had an eGFR below 60 mL/min/1.73 m^2^, and only 30% had evidence of micro or macroalbuminuria.

The baseline clinical characteristics in the studies focusing primarily on renal outcome [16-19] were also fairly similar to the CVOTs except for a considerable difference in baseline renal function. The EMPA-KIDNEY included patients with the lowest eGFR of 20 mL/min/1.73 m^2^. The mean eGFR ranged from 37 to 56 mL/min/1.73 m^2^, which was lower than in the CVOTs, and more than half of these patients showed evidence of either micro or macroalbuminuria. Despite differences in the study design and baseline patient characteristics, the findings for a reduction in risk for HHF were remarkably significant and consistent. However, patients' baseline characteristics seemed to influence the SGLT2 inhibitors’ benefit regarding the reduction in MACE. Trials that significantly reduced MACE were those with patients with a higher baseline CV risk and/or advanced kidney disease.

Clinical characteristics and patient demographics in the studies of secondary prevention of HF

The baseline characteristics are shown in Table 4 below.

**Table 4:** Patients’ clinical characteristics from studies on secondary prevention of heart failure T2DM: type 2 diabetes mellitus; NYHA: New York Heart Association; LVEF: left ventricle ejection fraction; NT-proBNP: N-terminal pro-B-type natriuretic peptide; AFib: atrial fibrillation; HF: heart failure; eGFR: estimated glomerular filtration; BP: blood pressure; ACE-I: angiotensin-converting enzyme inhibitor; ARB: angiotensin receptor blocker; ARNI: angiotensin receptor-neprilysin inhibitor; MRA: mineralocorticoid receptor antagonists.

Characteristics/demographics	SOLOIST WHF [18]	DAPA-HF [27]	EMPEROR REDUCED [28]	EMPEROR PRESERVED [29]	DELIVER [31]
Mean age (years)	69	66	66	71	71
Gender (males)	67.4%	76.2%	76.5%	55.4%	56.4%
Ethnicity					
White	93.3%	70%	71.1%	76.3%	70.7%
Blacks	4.1%	5.1%	6.6%	4.4%	2.6%
T2DM	100%	42%	50%	49%	45%
Non-diabetic		58%	50%	51%	55%
NYHA class					
Mean LVEF % (SD)	35 (median), IQR (28-47), LVEF <50% (79.1%)	31.2 (7)	27.7 (6), LVEF ≤30% (71.8%)	54.3 (9), LVEF >40-<50% (33.2%), LVEF ≥50-<60% (34.3%), LVEF ≥60% (32.5%)	54.0 (9), LVEF ≤49% (34.1%), LVEF 50-59% (36.2%) LVEF ≥60% (29.7%)
II	-	67.7%	75.1%	81.1%	73.9%
III	-	31.5%	24.4%	17.8%	25.8%
IV	-	0.8%	0.5%	0.3%	0.3%
NT-proBNP (median) IQR	1816.8 (854.7-3658)	1428 (857-2655)	1887 (1077-3429)	994 (501-1740)	729 (472-1299)
AFib/Flutter	-	-	-	-	1408 (956-2256)
HF etiology:					
Ischemic	N/A	55.5%	52.8%	36%	N/A
Non-ischemic	N/A	36.1%	47.2%	64%	N/A
Current worsening HF	100%	-	-	-	-
eGFR ml/min/1.73 m^2^ mean (SD)	Median 49.2 (39.5-61.2)	66.0 (20)	61.8 (22)	60.6 (20)	61 (19)
eGFR <60 ml/min/1.73 m^2^	N/A	40.6%	48%	50.2%	48.4%
Systolic BP mmHg (mean)	122 (111-135) (median)	122.0 ± 16.3	122.6 ± 15.9	131.8 ± 15.6	128 ± 15
HF medications					
ACE-I/ARB	82.1%	84.5%	70.5%	81%	72.7%
ARNI	15.3%	10.5%	18.3%	2.2%	5.3%
MRA	66.3%	71.5%	70.1%	37.3%	42.4%
Beta-blockers	92.8%	96%	94.7%	86.7%	82.8%
Diuretics	95.4%	93.4%	N/A	N/A	76.7%

The trials on HFrEF had more men and a relatively younger population compared to the trials on HFpEF. This was consistent with the epidemiology of HFpEF where advancing age and female sex are at increased risk for developing HFpEF [39]. All the trials included a majority of non-DM patients and chronic stable patients in the New York Heart Association (NYHA) functional class II except the SOLOIST-WHF study, where all the patients had T2DM and worsening HF.

Inclusion into any trial required obtaining a prespecified baseline natriuretic peptide level and measuring the LVEF by an echocardiogram. The minimum N-terminal pro-B-type natriuretic peptide (NT-proBNP) levels ranged from as low as 300 pg/ml in individuals in sinus rhythm in the EMPEROR-Preserved and the DELIVER trials to 5,000 pg/ml in individuals with atrial fibrillation or atrial flutter in the EMPEROR-Reduced trial. However, the median NT-proBNP across the trials ranged from as low as 974 pg/ml in the EMPEROR-Preserved trial to as high as 1,910 pg/ml in the EMPEROR-Reduced trial. The mean LVEF was as low as 27% in trials of HFrEF, with both trials on HFpEF having a mean LVEF of 54%. The EMPEROR trials included patients with an eGFR as low as 20 mL/min/1.73 m². The SOLOIST-WHF trial, however, had the lowest mean eGFR of approximately 50 mL/min/1.73 m², with all the other trials having a mean eGFR of about 60 mL/min/1.73 m² or slightly higher.

The patients in all the trials were on background guideline-directed medical therapy for HF. In particular, trials on HFrEF had more background use of angiotensin receptor-neprilysin inhibitors (ARNI) and mineralocorticoid receptor antagonists (MRA) than trials on HFpEF. Comparing the two main trials on HFrEF, it is notable that patients in the EMPEROR-Reduced trial were sicker than those in the DAPA-HF trial judging from their higher levels of NT-proBNP and much more reduced LVEF. Regarding the background use of HF therapy, more patients in the EMPEROR-Reduced were on an ARNI compared to those in the DAPA-HF trial. However, this did not affect the efficacy of the SGLT2 inhibitor in reducing the risk for HHF as both trials had very similar results. To further support these findings, Packer et al. [40] showed that the effect of an SGLT2 inhibitor in reducing the risk of CV death or HHF was independent of ARNI use.

The DELIVER trial had more patients in NYHA functional class III-IV and with higher NT-proBNP than in the EMPEROR-Preserved trial. This was perhaps because more patients in the DELIVER trial had a history of HHF (40%) compared to the EMPEROR-Preserved trial (23%). Another notable difference is that more patients in the EMPEROR-Preserved trial were on an angiotensin-converting enzyme inhibitor (ACE-I) or an angiotensin receptor blocker (ARB) compared to patients in the DELIVER trial.

The SOLOIST-WHF trial was in a unique care setting compared to all the other trials. It recruited only T2DM patients with worsening HF. Therefore, patients had a much higher median NT-proBNP (1,800) and a lower mean LVEF (35%). Additionally, the patients had the lowest mean eGFR out of all the trials (approximate eGFR of 50 mL/min/1.73 m²).

Safety

Data from studies on SGLT2 inhibitors show that these drugs are generally safe, well tolerated, and with an acceptable risk-benefit profile. The commonest adverse event reported in all trials was the small but increased risk for genitourinary infections. This includes urinary tract infections (UTIs) and genital mycotic infections. This risk is attributed to the increased amount of glucose in the urinary tract, which makes it a fertile environment for the growth of commensal microorganisms [41]. Regarding the increased risk of UTIs, the evidence was inconsistent [42]. The risk for genital mycotic infections with any SGLT2 inhibitors was consistent across trials and this risk is three-fold increased with any SGLT2 inhibitor use [40,41]. The most worrisome genital infection with rare cases reported is Fournier’s gangrene, and the US Food and Drug Administration (FDA) has included a black box warning about this risk [38,41].

Another serious but rare adverse event seen with SGLT2 inhibitors, particularly in DM patients, is diabetic ketoacidosis (DKA). Therefore, clinical conditions predisposing to an increase in risk should be evaluated for and managed [35,40]. SGLT2 inhibitors are associated with a small risk of hypoglycemia more so seen in T2DM patients on background glucose-lowering medications like insulin or sulfonylureas [40-42]. The risk is negligible in patients without DM [17,20,21].

Volume depletion resulting in hypotension is also a concern with SGLT2 inhibitor use because of their diuretic effect. However, no overt volume reduction was reported that resulted in a significantly increased risk for hypotension. This risk can, however, be reduced by dose adjustment of diuretic therapy. Acute kidney injury is a potential adverse event with SGLT2 inhibitors because of the hemodynamic changes that occur at the glomerular level [42]. Contrary to expectation, the renal outcomes trials with SGLT2 inhibitor demonstrated remarkable renoprotection and a decreased risk of acute renal injury [13,17,23]. The CANVAS program was the only trial that showed an elevated risk of lower limb amputation. This was observed to occur in individuals with a prior history of amputation or established peripheral artery disease [20].

Limitations and recommendations

Most of the data on the prevention of HF and the comparison between T2DM and non-DM patients came from a subgroup analysis, and this may have affected the power of the studies. It was also difficult to determine which phenotype of HF was prevented in the CVOTs and renal outcome trials because the definition of HF was not clearly defined. Currently, there is a paucity of data on the primary prevention of HF in patients with myocardial infarction, despite it being the leading cause of HF. Two major trials are ongoing in these cohorts, i.e., “Dapagliflozin Effects on Cardiovascular Events in Patients With an Acute Heart Attack (DAPA MI)” and “Empagliflozin in patients post myocardial infarction (EMPACT MI)” trials, which should give us more insights [41,42].

## Conclusions

In conclusion, SGLT2 inhibitors are effective for the primary and secondary prevention of HF across a broad spectrum of patient populations and care settings. The data have demonstrated that SGLT2 inhibitors reduce the risk of HF irrespective of diabetes or HF status and are generally safe with no clear evidence of any major safety concerns. We found that these medications are useful in both HFrEF and HFpEF irrespective of the diabetes status of a patient. These medications are generally well tolerated and are safe to use although there is a slightly increased risk of genitourinary infections and euglycemic ketoacidosis. Further randomized controlled trials would be useful to explore the efficacy and safety of these medications.
